# Lurasidone as add-on to fluoxetine in obsessive-compulsive disorder with comorbid restrictive anorexia: a case report

**DOI:** 10.1097/YIC.0000000000000502

**Published:** 2023-08-09

**Authors:** Laura Orsolini, Silvia Bellagamba, Umberto Volpe

**Affiliations:** Unit of Clinical Psychiatry, Department of Clinical Neurosciences/DIMSC, Polytechnic University of Marche, Ancona, Italy

**Keywords:** anorexia nervosa, augmentation strategy, eating disorder, lurasidone, obsessive-compulsive disorder, treatment resistant-OCD

## Abstract

Obsessive-compulsive disorder (OCD) is a pervasive disabling disorder that may overlap with other psychiatric conditions, including anorexia nervosa. Recent guidelines recommend low doses of second-generation antipsychotics as add-on therapy to selective serotonin reuptake inhibitors (SSRIs) for those patients presenting OCD who display residual symptomatology. Here we report a clinical case of a 45-years-old woman affected by severe OCD in comorbidity with anorexia nervosa, restrictive type (AN-r), treated with fluoxetine (titrated up to 40 mg/day) in augmentation with low doses of lurasidone (37 mg/day). At baseline and during a 6 months-follow-up we administered Clinical Global Impression-Severity, Symptom Checklist-90 items, Y-BOCS-II (Yale-Brown Obsessive Compulsive Scale) and EDI-3 (Eating Disorder Inventory). After 1 month of augmentation treatment, a clinically significant response was observed on obsessive symptoms at Y-BOCS-II (≥35% Y-BOCS reduction) and eating symptomatology at EDI-3. Full remission was reported after 3 months (Y-BOCS scoring ≤14) (*P* < 0.01). Further longitudinal and real-world effectiveness studies should be implemented to confirm these novel results, to investigate the potential of lurasidone as add-on strategy to SSRI in poor responder OCD patients, including treatment-resistant-OCD (tr-OCD), as well as in improving eating disorder symptomatology, whereas there is comorbidity with AN-r.

## Introduction

Obsessive-compulsive disorder (OCD) is a pervasive condition characterized by the presence of either obsessions (i.e. recurrent, and persistent thoughts, impulses, or images that are experienced as intrusive and inappropriate) and compulsions (i.e. repetitive behaviors that the individual needs to perform in response to obsessions, or according to rules that must be applied rigidly) (APA, 2022). OCD occurs in around 1% of the population worldwide (Ruscio *et al*., 2010), with a higher prevalence among males during childhood and among females in adolescence and adulthood (Mathes *et al*., 2019). In most cases, OCD can arise in comorbidity with other psychiatric disorders. Anorexia nervosa (AN) is commonly comorbid with OCD which is described in AN individuals with a prevalence rate of up to 62%; similarly, in around 5%-10% of OCD patients is described a comorbid lifetime history of AN (Brakoulias *et al*., 2017; Drakes *et al*., 2021; Riquin *et al.*, 2021; Sharma *et al*., 2021). In accordance with the epidemiological associations, clinical studies also described that AN and OCD share highly similar clinical phenotypes, such as perfectionism, rigidity, obsessive thoughts, persistence, and excessive ritual behaviors (Song *et al*., 2021). According to the most recent International Guidelines (Katzman *et al*., 2014; Janardhan Reddy *et al*., 2017; Riley *et al*., 2017; Menchon *et al*., 2019) cognitive-behavioral therapy (CBT) alone is recommended as an initial treatment in patients with mild-to-moderate OCD symptomatology, adding psychopharmacological treatment with selective serotonin reuptake inhibitors (SSRIs) as first-line treatment in more severe OCD. Nevertheless, a substantial percentage of patients still fail to respond to either CBT or SSRIs alone or combined in any meaningful way, or may display residual symptoms in 40–60% of cases, despite the optimization strategy (Skapinakis *et al*., 2016). For treatment resistant-OCD (TR-OCD) the American Psychiatric Association guidelines recommended a period of at least 8–12 weeks of SSRI treatment before considering a change in drug strategy and an addition of antipsychotics should be preferred over the switch to a different SSRI (Koran *et al*., 2007).

Hereby we describe the clinical case of a 45-year woman affected with a severe OCD and comorbid AN, restrictive type (AN-r), treated with fluoxetine in augmentation with low doses of lurasidone, followed up to 6 months. The full description of case-report has been provided, following the CARE (CAse REport) Statement, Checklist and Guidelines (Riley *et al*., 2017). The patient here described gave written consent for the publication of the presented findings.

## Case presentation

A 45-years-old Caucasian outpatient woman affected by a previously diagnosed severe OCD and AN-r consulted our Outpatient Psychiatric Services following the reoccurrence of an obsessive-compulsive symptomatology associated with anankastic thoughts and repetitive compulsions related to own home cleaning, control and hygiene, after the COVID-19 lockdown. She did not report any previous or current medical conditions. She denied usage of any other medication at the moment of her first evaluation. She did not report any tobacco or substance use. Her family history was negative for psychiatric conditions. At the first visit, she gave information about her personal life: she was living with her husband and her 12-years old daughter and she was working as a babysitter. The patient reported the onset of the eating disorder at 23 years old, with predominant restrictive behaviors associated with occasional bulimia. Minimal BMI when she was 23 years old was about 10 kg/m^2^. She early started psychopharmacological support and CBT psychotherapy with a gradual clinical remission of the eating disorder when she was 33 years old. However, at the age of 38, the emergence of obsessive thoughts occurred, especially referring to cleanliness, domestic order as well as contagion, with the forced need for accuracy and precision, accompanied by compulsions of cleaning the house and body to avoid contagion by germs. Consequently, she was referred to a psychiatrist who prescribed fluoxetine up to 40 mg daily and subsequently olanzapine up to 5 mg daily with a gradual full clinical remission after 5 months of treatment. She took psychotropic drugs for 2 years, then she decided to discontinue them due to pregnancy, despite that, she described good clinical stability for the following 2 years. However, during the first COVID-19 wave until March 2021, she displayed the recurrence of obsessive-compulsive symptomatology and the need to control her and her family’s food intake, particularly because she had obsession of contamination with stressful cleaning rituals concerning her daughter. She started to take fluoxetine at 20 mg daily dosage, after consultation with her previous psychiatrist, with a slight and unsatisfactory clinical response. At the moment of the visit in April 2021, the patient reported that in the previous 2 weeks, there would have been a flaring-up of concerns about body shapes and weight, with consequent restrictive behaviors and occasional binges followed by vomiting as compensatory strategies. Persistence and intensity of symptomatology lead the patient referring to our outpatient service at the University Hospital of Marche, in Ancona, Italy.

At the time of the first psychiatric evaluation, the patient described obsessions and compulsions as extremely distressing, time-consuming, and compromising social, family and work functioning resulting in avoidant behaviors. Therefore, the symptomatology referred by the patient met the DSM-5-TR criteria (APA, 2022) for an OCD [300.3; F42.0], through the use of the Mini International Neuropsychiatric Interview 5 interview (Rossi *et al*., 2004). A full clinical assessment for OCD, eating disorder and general psychopathology was administered at baseline and during the follow-up period, including the Italian version of the Yale-Brown Obsessive-Compulsive Scale (Y-BOCS-III) (Melli *et al*., 2015), the Italian version of the Eating Disorder Inventory-3 (Giannini *et al*., 2008), Clinical Global Impression Scale (Guy, 1976), Symptoms Checklist-90 (Prunas *et al*., 2012). Following a full clinical and laboratory assessment, considering her previous positive clinical response to fluoxetine, the same SSRI was initially confirmed with a gradual titration of up to 60 mg daily. However, the patient reported increased irritability, hyperactivity and alertness after increased dosage of fluoxetine. Therefore, considering the symptomatology reported, olanzapine was considered as an add-on strategy but the patient refused this drug because of the possibility of presenting side effects on weight gain. Therefore, the patient accepted an augmentation strategy with lurasidone that was gradually tapered up to 37 mg daily after dinner and melatonin 2 g daily before going to sleep for reported insomnia. A monthly follow-up visit was planned during which the same full clinical assessment was periodically administered: at the second follow-up visit (2 months after baseline), clinical response to obsessive thoughts and compulsions was observed while at the third follow-up visit, a full clinical remission was achieved and was maintained over the following 3 months (Table 1). Considering eating disorders symptoms, concerns about weight and body shape decreased after 1 month of therapy, while no variation was observed in BMI that remained at 18 kg/m^2^(mild thinness) (Table 1).

**Table 1:** Assessment scales results during 5 months follow up

	Y-BOCS scoring			CGI	SCL-90										EDI-3	Pharmaco therapy	BMI (Kg/m^2^)
	Obsessive symptoms	Compulsive symptoms	Total score		SOM	OC	INT	DEP	ANX	HOS	PHOB	PAR	PSY	GSI			
T0	23	21	44	5	1.1	2.3	2.4	2.8	1.7	1.7	1.1	2.5	1	1.9	Only GPMC	Fluoxetine 40–60 mg;	18
T1	18	18	36	5												Fluoxetine 60–40 mg; Lurasidone 37 mg	18
T2	10	9	19	2												Fluoxetine 40 mg; Lurasidone 37 mg	18
T3	6	5	11	2												Fluoxetine 40 mg; Lurasidone 37 mg	18
T4	4	4	8	1	0.5	1.6	1.6	2.2	1.2	1.2	0.6	0.7	0.8	0.1	Only GPMC	Fluoxetine 40 mg; Lurasidone 37 mg	18

T0, baseline; T1, 1 month from baseline; T2, 2 months from baseline; T3, 3 months from baseline; T4, 6 months from baseline.

## Discussion and conclusion

As previously reported, it was proposed to the patient an add-on strategy with lurasidone and fluoxetine, this pharmacotherapy was demonstrated to be safe and well tolerated by the patient, as already demonstrated by previous literature (Azhar and Shaban, 2021). Indeed, given that fluoxetine alone had poor effects on OC symptomatology and that in the previous episode of the disease the patient was already successfully treated through combination of fluoxetine and olanzapine, we considered add-on strategy with a second-generation antipsychotic drug. However, the patient refused olanzapine because of the possibility of metabolic side effects (this is the reason why we have also excluded risperidone), furthermore she refused to assume aripiprazole, too. For this reason, we considered an add-on strategy with lurasidone, mainly due to its relatively favorable pharmacodynamics profile associated with relatively low side effects. Even though pharmacokinetic inhibitory properties of fluoxetine on cytochrome CYP3A4 could theoretically influence hepatic biotransformation of lurasidone, real-world studies are needed to better establish drug-drug interaction between fluoxetine and lurasidone to identify dose correction regarding fluoxetine-lurasidone add-on therapy (Spina and de Leon, 2014). Indeed, the patient did not report any side effects and she also reported a subjective psychopathological improvement with a substantial increase in the perceived quality of life. Side effects were monitored through clinical interview and by administering specific rating scales, including the UKU side effect rating scale by using a retrospective chart review (Lingjærde *et al*., 1987).

Lurasidone is a second-generation antipsychotic that, to date, has indications for schizophrenia and depressive episodes associated with Bipolar I Disorder (bipolar depression), as monotherapy and as adjunctive therapy with lithium or valproate (FDA, 2023). Although lurasidone does not have an officially recognized therapeutic indication for the treatment of OCD, previous evidence described clinical cases of Bipolar Disorder patients with comorbid OCD-TR who successfully benefited from lurasidone treatment (Carmellini *et al*., 2019). From a pharmacodynamics perspective, lurasidone acts as full antagonist at dopamine D2 (with a dose-dependent D2 receptor occupancy; e.g., at the dosage of 40 mg/day is equal to 60–80%) and as antagonist of 5-HT2A and 5-HT7 receptors, and partial agonist of 5HT1A receptor (Orzelska-Górka *et al*., 2022). Moreover, compared to other second-generation antipsychotics, lurasidone displays the highest binding affinity for the 5-HT7 receptor, which may explain its pro-cognitive and mood-stabilizing effects (Jaeschke *et al*., 2016). In addition, lurasidone acts as an antidepressant by increasing dopamine activity in the prefrontal cortex, through its effect on 5-HT2A and 5-HT1A receptors (Bawa and Scarff, 2015) and increasing cortical serotonin levels by blocking 5-HT2A receptors (Fornaro *et al*., 2017). Furthermore, lurasidone displays a low activity at α1 and α2-adrenergic receptors, histaminergic H1, and muscarinic receptors with consequent low risk to determine orthostatic hypotension, sedation, weight gain and cognitive impairment (Citrome *et al*., 2014). OCD pato-physiology has been widely studied, and genetic and neuroimaging studies suggested an altered cortico-striato-thalamo-cortical circuit with the involvement of three neurotransmitter systems: serotonin, dopamine and glutamate (Pauls *et al*., 2014). Augmentation strategy with second-generation antipsychotics showed to be effective in addition to SSRIs, due to their activity at both D2 receptors and antagonism at 5-HT2A receptors (Pittenger, 2021). While there is an overall agreement on introducing an add-on risperidone and/or aripiprazole to SSRIs in OCD (Kim *et al*., 2018), there is still no data for lurasidone (Azhar and Shaban, 2021).

Despite these promising clinical findings, our clinical case has some limitations that should be described. First, the nature of the study being an isolated case report could limit the generalizability of our findings to larger clinical sample. Further, both randomized clinical trials as well as larger cohort studies should be carried out in real-world settings to evaluate, respectively, the efficacy and the effectiveness of lurasidone-fluoxetine add-on strategy in OCD individuals with and/or without a comorbid AN-r. Second, these preliminary findings should be confirmed by longitudinally monitoring obsessive and compulsive symptomatology over the time, in order to evaluate whether the measured efficacy and tolerability could be maintained over the time, also in terms of functionality. Overall, our clinical case report could significantly help clinicians in considering different augmentation alternative strategies for the management of OCD in comorbidity with AN-r and evaluating if lurasidone may represent an add-on strategy to SSRIs also in those more severe OCD cases not necessarily in comorbidity with an AN-r. Further studies are needed to confirm these preliminary findings.

## Acknowledgements

Conceptualization, LO; methodology, L.O.; formal analysis, S.B.; data curation, S.B. and L.O.; writing-original draft preparation, S.B. and L.O., writing-review and editing, L.O.; supervision, U.V.. All authors have read and agreed to the published version of the manuscript.

Informed consent statement: Written informed consent has been obtained from the patient to publish this paper.

The statements, opinions and data contained in all publications are solely those of the individual author(s) and contributor(s) and not of International Clinical Psychopharmacology and/or the editor(s). International Clinical Psychopharmacology and/or the editor(s) disclaim responsibility for any injury to people or property resulting from any ideas, methods, instructions or products referred to in the content.

### Conflicts of interest

There are no conflicts of interest.
